# Spatiotemporal Analysis of Dengue Incidence and Associated Factors: An Ecological Study Across Municipalities in Bahia, Brazil (2019-2024)

**DOI:** 10.7759/cureus.116934

**Published:** 2026-09-25

**Authors:** Thailany A Magalhães, Severino S Agra Filho, Patricia C Borja

**Affiliations:** 1 Department of Environmental Engineering, Universidade Federal da Bahia, Salvador, BRA

**Keywords:** brazil, dengue fever, geospatial analysis, public health, spatial autocorrelation, spatial regression

## Abstract

Introduction: Dengue remains a major public health challenge in Brazil, characterized by substantial spatial and temporal heterogeneity in transmission. This study aimed to analyze the spatiotemporal characteristics of dengue incidence across the municipalities of Bahia, Brazil, throughout the study period and to identify socioeconomic, demographic, and urban infrastructure factors associated with dengue incidence in the years with the lowest and highest state-level incidence.

Methods: An ecological study was conducted using probable dengue cases reported in Bahia from 2019 to 2024. The complete six-year series was analyzed descriptively to characterize temporal variation and the spatial distribution of dengue incidence across 417 municipalities. Incidence rates per 100,000 inhabitants were smoothed using a local empirical Bayes estimator. Spatial autocorrelation was assessed annually using Global Moran’s I and Local Indicators of Spatial Association (LISA). Ordinary least squares (OLS), spatial autoregressive (SAR), and spatial error models (SEMs) were fitted for 2021 and 2024, the years with the lowest and highest state-level incidence, respectively, to contrast low- and high-transmission contexts. Socioeconomic, demographic, and urban infrastructure indicators from the 2010 Census and 2020 MUNIC (Municipal Basic Information Survey) were treated as time-invariant in both regression models.

Results: Between 2019 and 2024, 506,792 probable dengue cases were reported across the municipalities of Bahia. The incidence was lowest in 2021 (166.6 cases per 100,000 inhabitants) and highest in 2024 (1,558.7 cases per 100,000 inhabitants). Significant positive spatial autocorrelation was observed in all years, with high-high clusters shifting from the Far West region in 2021 to the South-Central region in 2024. SAR showed the best fit. Per capita household income was positively associated with incidence in 2021 (β=0.003; 95% CI: 0.001-0.005) and 2024 (β=0.002; 95% CI: 0.000-0.003). In 2024, sewerage coverage (β=-0.006; 95% CI: -0.011 to -0.001) and the proportion of the population classified as Black or mixed-race (β=-0.020; 95% CI: -0.032 to -0.008) were negatively associated with incidence.

Conclusions: The findings demonstrate marked spatiotemporal heterogeneity and spatial clustering of dengue incidence in Bahia and highlight the relevance of socioeconomic, demographic, and urban infrastructure conditions to its spatial distribution. The findings provide geospatial evidence to support geographically targeted dengue surveillance and control strategies.

## Introduction

Dengue is an important mosquito-borne viral disease affecting tropical and subtropical regions and remains a major public health challenge. Its transmission is influenced by the interaction of environmental, demographic, socioeconomic, and urban factors, which may vary substantially across territories and contribute to spatial differences in disease incidence [1-3]. In Brazil, dengue has expanded considerably over recent decades, with recurrent epidemics and widespread territorial transmission [4-6]. Its distribution has been associated with environmental and urban conditions, socioeconomic inequalities, and limitations in basic sanitation infrastructure. These factors may contribute to heterogeneous transmission patterns across municipalities and require approaches capable of accounting for the spatial context of disease occurrence [3,7].

In Brazil, the state of Bahia is a relevant setting for investigating this territorial heterogeneity. With approximately 14 million inhabitants distributed across 417 municipalities, the state presents marked socioeconomic, demographic, and urban infrastructure inequalities and persistent dengue transmission. Previous studies have identified associations between dengue occurrence and climatic, socioeconomic, and territorial characteristics in different areas of Bahia. However, investigations integrating spatial analysis with socioeconomic, demographic, and urban infrastructure indicators at the municipal level remain limited [7-11]. This gap is relevant because identifying spatial patterns and territorial characteristics associated with dengue incidence can contribute to a more geographically targeted understanding of transmission and support the prioritization of surveillance and control measures.

Spatial analytical methods provide an appropriate framework for investigating the geographic distribution of dengue, identifying nonrandom spatial patterns, and assessing spatial dependence between neighboring areas. Global and local spatial autocorrelation analyses can identify high-incidence clusters, while spatial regression models can account for spatial dependence when examining associations between dengue incidence and territorial characteristics [9,12-14]. These approaches can provide geographically explicit evidence to support disease surveillance and control.

Given the marked temporal and territorial variability of dengue transmission, examining a period that encompasses contrasting epidemiological contexts is important for understanding how spatial patterns and their associated territorial characteristics change over time. The 2019-2024 period includes years of relatively high transmission, the COVID-19 pandemic period, and the major epidemic observed in 2024. The study aimed to analyze the spatiotemporal characteristics of dengue incidence across the municipalities of Bahia, Brazil, throughout the study period and to identify socioeconomic, demographic, and urban infrastructure factors associated with dengue incidence in the years with the lowest and highest state-level incidence.

Based on previous evidence linking dengue transmission to urban infrastructure, population concentration, and socioeconomic conditions [3,7,12,13], we expected greater sewerage and water-supply coverage to be associated with lower dengue incidence, whereas higher population density, the presence of slums, and flood occurrence were expected to be associated with higher incidence. For socioeconomic indicators such as household income and racial composition, we did not assume a uniform direction of association because their relationships with reported dengue incidence may be mediated by differences in health care access, diagnosis, surveillance, and other territorial conditions.

## Materials and methods

Study design and area

An ecological epidemiological study was conducted. The study area was the state of Bahia, located in the Northeast region of Brazil, with a territorial area of 564,760 km² and a population of 14,141,626 inhabitants. The units of analysis were the 417 municipalities of the state.

Epidemiological data

Records of probable dengue cases, according to the clinical-epidemiological criteria established by the Brazilian Ministry of Health and reported through the Notifiable Diseases Information System (SINAN), were used for the period from 2019 to 2024. This period was selected because it encompasses years of low and high transmission, including the COVID-19 pandemic period. For each municipality and year within the study period, dengue incidence rates per 100,000 inhabitants were calculated using population estimates provided by the Brazilian Institute of Geography and Statistics (IBGE).

Socioeconomic, demographic, and sanitation data

Independent variables were obtained from the 2010 IBGE Demographic Census and the 2020 Municipal Basic Information Survey (MUNIC). The 2010 Demographic Census was used because more recent census data with municipal-level disaggregation were not available during the study period. Independent variables were selected based on literature evidence indicating the influence of socioeconomic, demographic, and urban infrastructure factors on dengue transmission dynamics. A set of indicators representing these different dimensions was selected for the final analysis while avoiding explanatory redundancy and ensuring the stability of the model estimates.

The following variables were selected: population density (inhabitants per km²); percentage of households with water supplied through the public water-supply network; percentage of households connected to the sewerage network; percentage of the female population; percentage of the population classified as Black or mixed-race, aggregating the IBGE categories Black (preto) and mixed-race (pardo); and per capita household income. The occurrence of floods and the presence of slums were considered dichotomous variables (yes/no), based on information from MUNIC.

Because these indicators were derived from a single time point for each data source (2010 Census and 2020 MUNIC), identical covariate values were used in the 2021 and 2024 regression models; these variables did not vary across years in the present analysis. Consequently, differences in statistical significance or estimated associations between the two regression years cannot be attributed to temporal changes in the exposure variables but should instead be interpreted in relation to differences in the magnitude, distribution, and spatial configuration of dengue incidence across years.

Spatial and statistical analysis

Probable dengue cases were aggregated at the municipal level using the 2021 Digital Municipal Map of the Political-Administrative Division of the State of Bahia. All municipalities, including those with zero reported dengue cases, were retained in the spatial analysis. Missing municipal case counts in the original database were systematically identified and cross-referenced with SINAN surveillance records. When SINAN records confirmed dengue cases for municipalities with missing values, the corresponding case counts were corrected using the surveillance data. Remaining missing values, confirmed as zero in the SINAN records, were recorded as zero. This validation procedure ensured a complete municipal dataset before incidence-rate calculation and subsequent analyses. For the independent variables, all selected demographic and infrastructure indicators from the 2010 Census and 2020 MUNIC were complete for the 417 municipalities; no imputation or case exclusion was required.

To reduce instability in incidence rates in municipalities with small populations, local empirical Bayes smoothing was applied. The procedure was implemented using the EBlocal function from the spdep package, with the annual number of dengue cases as the numerator and the corresponding municipal population as the denominator. The neighborhood structure used for smoothing was defined using first-order Queen polygon contiguity, in which municipalities sharing either a boundary or a vertex were considered neighbors. The resulting empirical Bayes estimates were expressed per 100,000 inhabitants.

Spatial dependence was assessed using Global Moran’s I and Local Indicators of Spatial Association (LISA). The LISA results were used to classify municipalities according to four types of spatial association: High-High and Low-Low, representing positive spatial association between municipalities and their neighbors, and High-Low and Low-High, representing spatial outliers in which municipalities had values that differed from those of their neighbors. A first-order Queen contiguity structure was used to define neighboring municipalities. The binary neighborhood matrix was subsequently converted to row-standardized spatial weights before the spatial autocorrelation and regression analyses. Statistical significance was assessed using 999 permutations and a significance level of 5%.

To examine the association between dengue incidence and the independent variables, an ordinary least squares (OLS) multiple linear regression model was initially fitted. The dependent variable was the smoothed dengue incidence rate after natural logarithmic transformation, calculated as ln(incidence + 1) to accommodate municipalities with zero cases. The log-linear approach was selected because the dependent variable, dengue incidence rate, is a continuous epidemiological measure. The application of the local empirical Bayes estimator further stabilizes municipal incidence rates, particularly in municipalities with small populations, thereby mitigating small-area instability. Consequently, the natural logarithmic transformation was applied to better satisfy the assumptions of normality and homoscedasticity required for Gaussian-based spatial autoregressive (SAR) and spatial error models (SEMs). This analytical workflow is a robust and widely used approach in ecological studies investigating the spatial dynamics of arboviruses at the municipal level in Brazil [9,12,13].

Based on the time series of dengue incidence, a cross-sectional spatial regression approach was deliberately chosen to evaluate the years with the lowest (2021) and highest (2024) state-level incidence rates. Rather than modeling the continuous temporal trend across the entire study period, this design maximizes epidemiological contrast, enabling an in-depth comparative analysis of the spatial determinants of dengue under two markedly different contexts: a period of low transmission and an epidemic peak. Furthermore, because the socioeconomic and urban infrastructure indicators are time-invariant at the municipal level (2010 Census and 2020 MUNIC), this comparative cross-sectional design is well suited to assess how the same territorial characteristics are associated with disease incidence under varying magnitudes of epidemiological pressure.

Because the dependent variable was log-transformed, regression coefficients (β) were exponentiated to express associations as the percentage change in incidence associated with a one-unit increase in continuous independent variables or a change from the reference category for binary variables, holding other variables constant, according to the following formula:

$\%\Delta= (e^{\beta} - 1) \times 100$

Model adequacy was assessed using tests for multicollinearity, heteroscedasticity, residual normality, and spatial autocorrelation. Multicollinearity was evaluated using the variance inflation factor (VIF), with values below 5 considered acceptable. Heteroscedasticity was assessed using the White test, residual normality using the Jarque-Bera test, and spatial autocorrelation of model residuals using Global Moran's I.

In the presence of spatial dependence, spatial regression models were fitted, including the SAR and SEM. Model performance was compared using log-likelihood, Akaike information criterion (AIC), Schwarz Bayesian information criterion (BIC), adjusted R², and residual spatial autocorrelation [12].

All analyses were conducted using R software, version 4.3.1 (R Foundation for Statistical Computing), an open-source statistical computing environment. The main R packages used for spatial autocorrelation and spatial regression analyses were spdep and spatialreg.

Ethical considerations

This study used only secondary, aggregated, and publicly available data, with no possibility of individual participant identification. In accordance with Resolution No. 466/2012 of the Brazilian National Health Council, review by a Research Ethics Committee was not required.

## Results

From 2019 to 2024, a total of 506,792 probable dengue cases were recorded in Bahia, Brazil. The temporal distribution showed marked variation, with higher numbers of reported cases in 2019, 2020, and 2024. The highest state-level crude incidence rate was observed in 2024, at 1,558.7 cases per 100,000 inhabitants, whereas the lowest rate occurred in 2021, at 166.6 cases per 100,000 inhabitants. Incidence subsequently increased in 2022 and 2023 (Figure 1).

**Figure 1:**
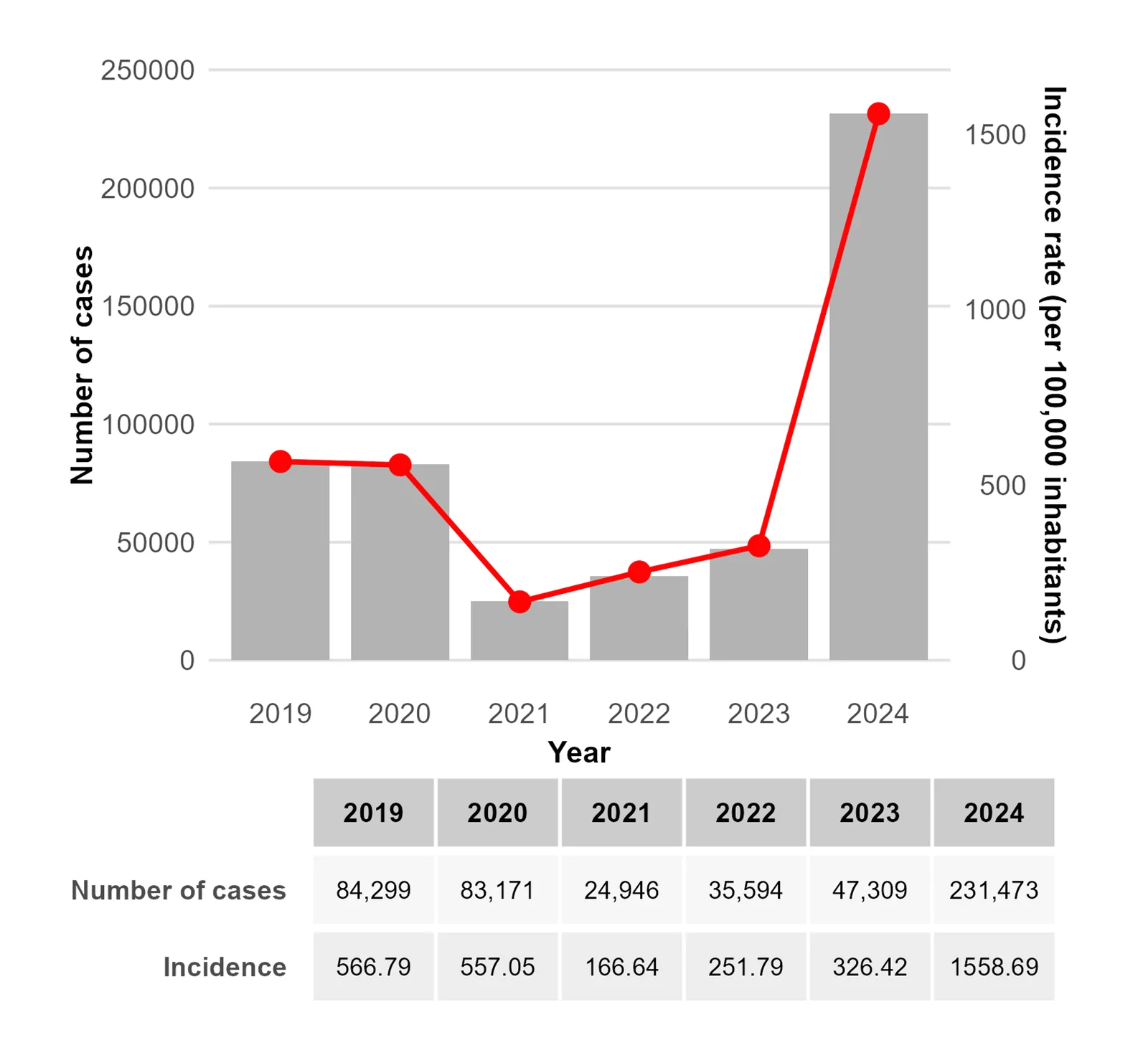
Number of dengue cases and incidence rate, Bahia, 2019-2024 (n=417)

The number of municipalities reporting dengue cases ranged from 308 in 2021 to 415 in 2024. In absolute numbers, Salvador (48,171 cases), Vitória da Conquista (42,839 cases), and Feira de Santana (28,110 cases) recorded the highest numbers of reported cases during the study period.

Municipal-level descriptive statistics for empirical Bayes-smoothed dengue incidence rates are presented in Table 1. Across the study period, the distribution of smoothed incidence rates was right-skewed, with means consistently exceeding medians and with substantial dispersion and wide upper ranges. The median smoothed incidence was lowest in 2021 (20.3 cases per 100,000 inhabitants; IQR: 7.4-64.9) and highest in 2024 (785.0 cases per 100,000 inhabitants; IQR: 301.0-2,107.0). The number of municipalities with zero reported cases before smoothing ranged from 2 (0.5%) in 2024 to 109 (26.1%) in 2021. The pronounced right-skewness of the smoothed incidence distribution supported the subsequent logarithmic transformation used in the spatial regression models.

**Table 1:** Descriptive statistics of smoothed municipal dengue incidence rates and number of municipalities with zero reported cases, Bahia, Brazil, 2019-2024 (n=417) Note: Incidence rates are expressed per 100,000 inhabitants. Mean, median, quartiles, minimum, maximum, and SD refer to local empirical Bayes-smoothed municipal incidence rates. The number of municipalities with zero cases refers to crude reported dengue case counts before smoothing. SD, standard deviation

Year	Mean	Median	IQR (Q1-Q3)	Minimum	Maximum	SD	Municipalities with zero reported cases, n (%)
2019	412.0	146.0	42.5-474.0	0.02	4,135.0	641.0	56 (13.4%)
2020	621.0	319.0	129.0-753.0	0.10	6,991.0	823.0	20 (4.8%)
2021	170.0	20.3	7.4-64.9	0.001	3,908.0	439.0	109 (26.1%)
2022	302.0	57.8	12.6-269.0	0.006	5,284.0	654.0	85 (20.4%)
2023	280.0	76.2	23.4-255.0	0.003	5,474.0	611.0	45 (10.8%)
2024	1,735.0	785.0	301.0-2,107.0	8.13	20,970.0	2,462.0	2 (0.5%)

The spatial distribution of dengue incidence rates, smoothed using the local empirical Bayes estimator, varied substantially among municipalities in Bahia. The highest smoothed rates were concentrated mainly in the Far West, North-Central, and São Francisco Valley regions (Figure 2).

**Figure 2:**
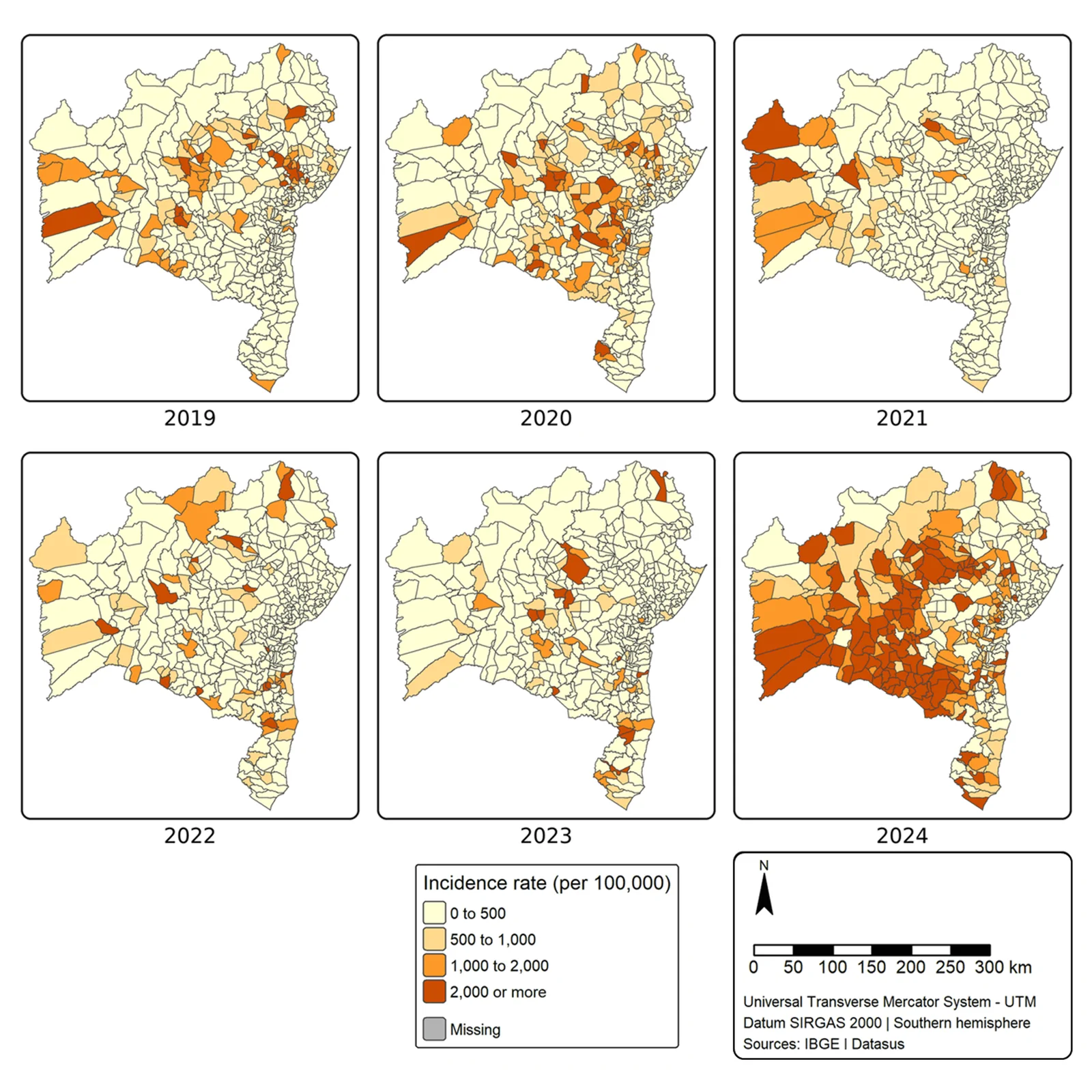
Spatial distribution of the smoothed dengue incidence rate by municipality, Bahia, 2019-2024 (n=417) IBGE, Brazilian Institute of Geography and Statistics

Global Moran’s I indicated significant positive spatial autocorrelation in dengue incidence in all study years (Table 2). The highest value was observed in 2021 (I=0.412, p<0.001), followed by 2024 (I=0.393, p<0.001) and 2019 (I=0.285, p<0.001) (Table 2).

**Table 2:** Global Moran's I values for dengue incidence rates recorded in Bahia municipalities, 2019-2024

Year	Moran's I	p-value
2019	0.285	0.001
2020	0.173	0.001
2021	0.412	0.001
2022	0.155	0.001
2023	0.103	0.003
2024	0.393	0.001

The LISA analysis identified significant local spatial associations in each study year (Table 3). High-High clusters, representing municipalities with high incidence rates surrounded by neighbors with similarly high rates, ranged from 9 municipalities (2.2%) in 2023 to 41 (9.8%) in 2024. Low-Low clusters, representing municipalities with low incidence rates surrounded by neighbors with similarly low rates, were the most frequent category, ranging from 32 municipalities (7.7%) in 2020 to 84 (20.1%) in 2022. High-Low and Low-High outliers were less common, ranging from 0 to 10 municipalities and 2 to 17 municipalities, respectively, across the study period.

**Table 3:** Distribution of municipalities by LISA quadrant, Bahia, 2019-2024 (n=417) LISA, Local Indicators of Spatial Association

Year	High-High (n, %)	Low-Low (n, %)	High-Low (n, %)	Low-High (n, %)	Not significant (n, %)
2019	37 (8.9%)	75 (18.0%)	1 (0.2%)	13 (3.1%)	291 (69.8%)
2020	23 (5.5%)	32 (7.7%)	8 (1.9%)	15 (3.6%)	339 (81.3%)
2021	36 (8.6%)	80 (19.2%)	0 (0%)	4 (1.0%)	297 (71.2%)
2022	17 (4.1%)	84 (20.1%)	2 (0.5%)	6 (1.4%)	308 (73.9%)
2023	9 (2.2%)	56 (13.4%)	3 (0.7%)	17 (4.1%)	332 (79.6%)
2024	41 (9.8%)	76 (18.2%)	10 (2.4%)	2 (0.5%)	288 (69.1%)

The spatial configuration of local clusters varied substantially across the study period (Figure 3). High-High clusters showed different patterns of spatial concentration and dispersion over time. In 2019, High-High municipalities were distributed across several areas of the state, whereas in 2020 they appeared as smaller and more dispersed groups. In 2021, the High-High pattern became markedly concentrated in the western portion of Bahia, forming a large contiguous cluster in the Far West region. In 2022 and 2023, High-High clusters were fewer and more spatially localized, with concentrations primarily in the eastern and southern portions of the state. In 2024, the number of High-High municipalities increased to 41 (9.8%), with clusters concentrated mainly in the South-Central region and in portions of the eastern part of the state. Low-Low clusters were also spatially heterogeneous but remained particularly prominent in 2021, 2022, and 2024, indicating persistent areas of low dengue incidence surrounded by municipalities with similarly low incidence.

**Figure 3:**
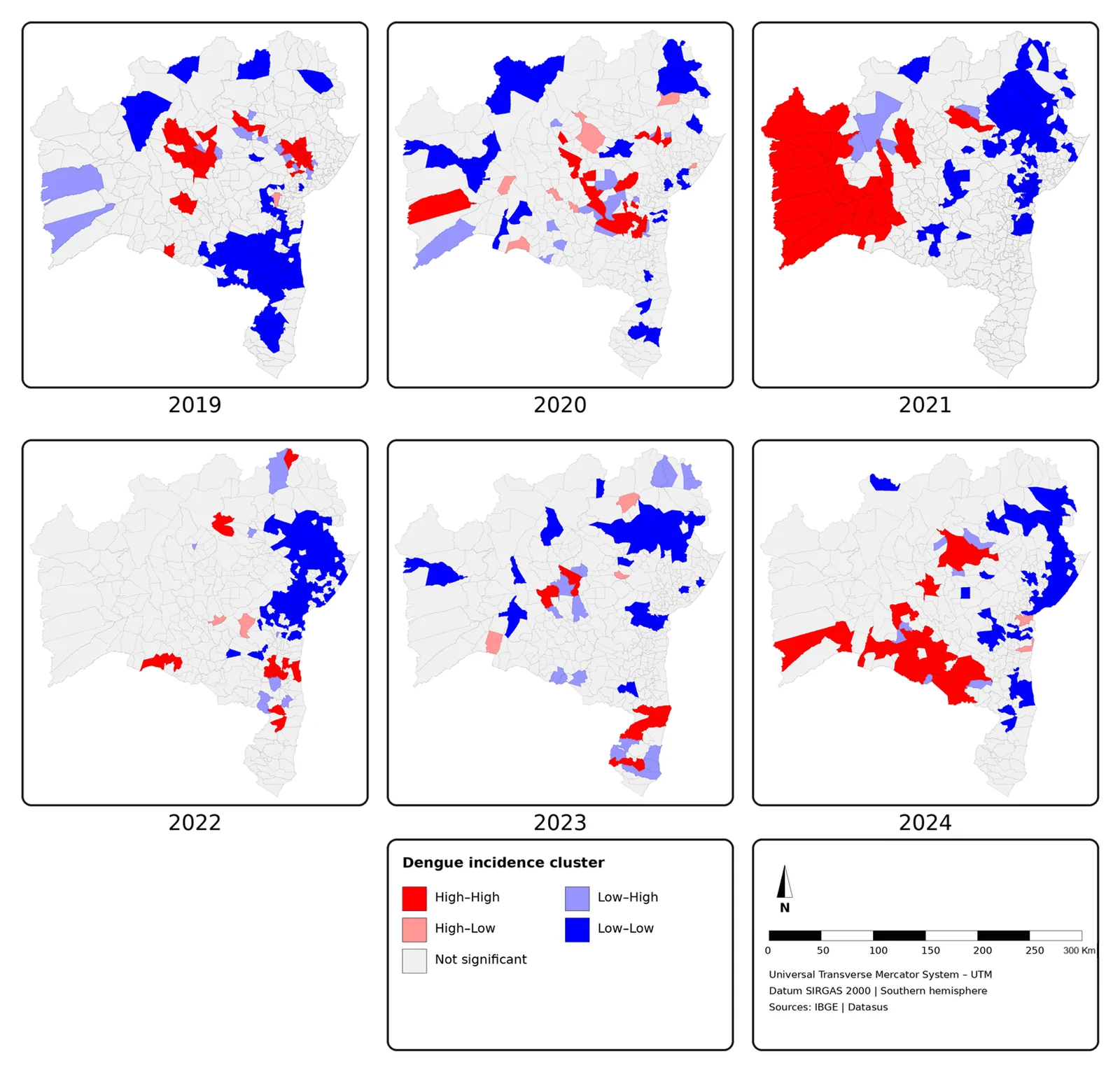
Local spatial clusters of dengue incidence according to the Local Moran's I index, by municipality, Bahia, 2019-2024 (n=417) IBGE, Brazilian Institute of Geography and Statistics

Based on the temporal distribution of dengue incidence, 2021 and 2024 were selected for spatial regression analysis because they represented the years with the lowest and highest state-level incidence rates, respectively. OLS regression models were initially fitted using the natural logarithm of the smoothed dengue incidence rate as the dependent variable.

The OLS model showed a coefficient of determination (R²) of 0.101 in 2021 and 0.178 in 2024. In 2021, the residuals did not meet the assumptions of normality and homoscedasticity, and a robust covariance matrix correction was therefore applied. In 2024, the assumptions of normality and homoscedasticity were met. In both years, no evidence of relevant multicollinearity was observed among the independent variables, with VIF values below 5. The OLS residuals showed statistically significant spatial autocorrelation in both years, with Moran's I values of 0.426 (p<0.001) in 2021 and 0.308 (p<0.001) in 2024. Spatial regression models were therefore fitted to account for the spatial dependence identified in the OLS residuals (Table 4).

**Table 4:** Model fit and diagnostic statistics for OLS, SAR, and SEM regressions of dengue incidence in Bahia, Brazil, 2021 and 2024 (n=417) *p<0.001 for residual spatial autocorrelation. OLS, ordinary least squares; SAR, spatial autoregressive model; SEM, spatial error model; AIC, Akaike information criterion; BIC, Schwarz Bayesian information criterion

Indicator	2021			2024		
	OLS	SAR	SEM	OLS	SAR	SEM
R²	0.101	0.421	0.0536	0.178	0.42	0.163
Log-likelihood	-832.297	-730.171	-730.590	-681.013	-631.294	-635.177
AIC	1,659.85	1,482.34	1,483.18	1,382.03	1,284.59	1,292.35
BIC	1,700.18	1,526.71	1,527.54	1,422.36	1,328.95	1,336.72
Residual Moran's I	0.426*	-0.037	-0.026	0.308*	-0.040	-0.039

Both the SAR model and SEM substantially reduced residual spatial autocorrelation. Neither the SAR model nor the SEM showed statistically significant residual spatial autocorrelation. In both years, the spatial models outperformed the OLS model, showing higher log-likelihood values and lower AIC and BIC values. The SAR model showed the best overall fit in both years and was therefore selected for the interpretation of factors associated with dengue incidence (Table 4).

In the 2021 SAR model, per capita household income was positively associated with dengue incidence (β=0.003; 95% CI, 0.001 to 0.005; p=0.002), corresponding to an estimated 0.29% increase in dengue incidence for each one-unit increase in per capita household income. No other independent variable showed a statistically significant association with dengue incidence (Table 5).

**Table 5:** Regression coefficients (β), 95% CI, p-values, and approximate percentage change in incidence (%Δ) from SAR models of dengue incidence according to socioeconomic, demographic, and infrastructure variables in Bahia, Brazil, 2021 and 2024 (n=417) β, regression coefficient; %Δ, approximate percentage change in incidence; SAR, spatial autoregressive model

Independent variables	2021 β	2021 95% CI	2021 p	2021 %Δ	2024 β	2024 95% CI	2024 p	2024 %Δ
Water supply network coverage (%)	0.005	-0.004; 0.013	0.284	0.47%	-0.003	-0.010; 0.004	0.470	-0.26%
Sewerage network (%)	-0.001	-0.008; 0.005	0.646	-0.14%	-0.006	-0.011; -0.001	0.020	-0.59%
Slums	0.279	-0.003; 0.561	0.053	32.18%	0.191	-0.037; 0.419	0.100	21.06%
Flooding	-0.104	-0.353; 0.145	0.412	-9.89%	0.011	-0.190; 0.212	0.917	1.07%
Population density	-0.001	-0.001; 0.000	0.067	-0.06%	-0.000	-0.001; 0.000	0.134	-0.04%
Female population (%)	-0.083	-0.194; 0.028	0.144	-7.94%	0.063	-0.026; 0.152	0.167	6.50%
Race/skin color (%)	-0.003	-0.018; 0.012	0.686	-0.30%	-0.020	-0.032; -0.008	0.001	-1.98%
Per capita household income	0.003	0.001; 0.005	0.002	0.29%	0.002	0.000; 0.003	0.018	0.18%

In the 2024 SAR model, dengue incidence was negatively associated with the proportion of households connected to a sewerage network (β=-0.006; 95% CI, -0.011 to -0.001; p=0.020), corresponding to an estimated 0.59% decrease in dengue incidence for each percentage-point increase in sewerage coverage, and with the proportion of the population classified as Black or mixed-race (β=-0.020; 95% CI, -0.032 to -0.008; p=0.001), corresponding to an estimated 1.98% decrease for each percentage-point increase in this population proportion. Per capita household income was again positively associated with dengue incidence (β=0.002; 95% CI, 0.000 to 0.003; p=0.018), corresponding to an estimated 0.18% increase in dengue incidence for each one-unit increase in per capita household income (Table 5).

## Discussion

The findings of this study demonstrate marked spatial and temporal heterogeneity in dengue incidence across the municipalities of Bahia, with significant positive global spatial autocorrelation observed throughout the study period. The identification of High-High spatial clusters indicates that dengue incidence was not randomly distributed across the state, but rather exhibited geographically structured patterns that varied over time. The persistence of significant spatial dependence also supports the use of spatial regression approaches when examining associations between dengue incidence and territorial characteristics.

The reduction in incidence rates observed in 2021 coincided with the most intense period of the COVID-19 pandemic in Brazil, which was characterized by substantial reorganization of health services and changes in patterns of health care-seeking behavior [15,16]. Previous studies have reported reductions in the use of health services for conditions unrelated to COVID-19 and changes in surveillance activities during this period, which may have affected the detection and notification of dengue cases [16,17]. Similarly, the temporal pattern observed in 2020 was atypical, with a decline in reported cases after March, coinciding with the implementation of social distancing measures, a pattern that partially extended into the following year [16,17]. These findings should therefore be interpreted in the context of possible changes in health care access, case detection, and disease surveillance during the pandemic.

In contrast, the highest rates recorded in 2024 occurred in a context of atypical climatic conditions, particularly associated with the El Niño event, which was related to anomalous temperature and precipitation patterns. Evidence has indicated that temperature, precipitation, and humidity patterns can create environmental conditions more favorable to *Aedes aegypti* circulation and viral transmission in certain regions, especially those historically characterized by higher vector burden [18,19]. However, climatic variables were not directly evaluated in the present study. These factors should therefore be considered as part of the broader explanatory context for the 2024 increase, rather than as determinants demonstrated by the present analysis.

The spatial clusters identified in the Far West in 2021 and in the South-Central region in 2024 indicate that dengue transmission was not confined to a single persistent geographic area, but rather shifted in location and extent over the study period. This spatial variability suggests the relevance of territorial and structural factors that may differ across regions and over time. Previous studies have indicated that parts of Bahia face inequalities in access to basic sanitation and regular water supply, conditions that may create environmental contexts more favorable to the maintenance of vector breeding sites [20]. The frequent practice of household water storage in these municipalities, resulting from deficiencies in water supply, may further increase the availability of potential breeding sites for *Aedes aegypti* [20,21].

Spatial modeling indicated that socioeconomic and demographic variables exhibited complex associations with dengue incidence at the municipal level, which varied according to the year analyzed. The positive association between per capita household income and dengue incidence, observed in both analyzed years (an estimated 0.29% and 0.18% increase in incidence per unit increase in income in 2021 and 2024, respectively), has been discussed in the literature as a possible indicator of reporting bias. Municipalities with better socioeconomic conditions tend to have greater access to health services and surveillance systems, which may result in a higher probability of case diagnosis and registration, without necessarily reflecting a higher risk of illness [13,22,23]. However, the present ecological design does not allow this mechanism to be directly evaluated, and differences in actual exposure to dengue cannot be excluded.

The fact that sewerage coverage and the proportion of the population classified as Black or mixed-race were significantly associated with dengue incidence in 2024, but not in 2021, should be interpreted cautiously. Because the same municipal covariates derived from the 2010 Census and 2020 MUNIC were used in both regression models, this difference does not indicate temporal changes in the exposures themselves. Rather, it may reflect differences in the magnitude and spatial distribution of dengue incidence between the two years, including the shift in High-High clusters from the Far West in 2021 to the South-Central region in 2024. The substantially lower-incidence context in 2021, together with the larger number of municipalities with zero reported cases before smoothing (109, compared with 2 in 2024), may also have influenced the strength and detectability of associations with territorial characteristics. In addition, the 2021 incidence estimates should be interpreted in light of possible disruptions to case detection and notification during the COVID-19 pandemic. Therefore, these year-specific associations should not be interpreted as evidence that the underlying territorial determinants changed over time, but rather as context-dependent ecological associations observed under different epidemiological and surveillance conditions.

In 2024, greater sewerage network coverage was negatively associated with dengue incidence, with each percentage-point increase in coverage associated with an estimated 0.59% reduction in incidence. This finding is consistent with previous evidence indicating that sanitation infrastructure represents an important structural component of the territorial context in which dengue transmission occurs [12,24]. Municipalities with lower sewerage coverage may experience environmental conditions more favorable to vector proliferation. However, sewerage coverage may also function as a broader marker of urban infrastructure and socioeconomic conditions, and the present analysis cannot determine whether the observed association reflects a direct effect of sanitation infrastructure or other correlated territorial characteristics.

The inverse association between the proportion of the population classified as Black or mixed-race and dengue incidence observed in 2024 (an estimated 1.98% reduction in incidence per percentage-point increase) should be interpreted cautiously within the context of the social and structural inequalities that shape access to health care, diagnosis, surveillance, and environmental conditions in Brazil. This finding should not be understood as indicative of biological differences in susceptibility, but rather as a reflection of social processes that influence access to health care, diagnosis, and reporting [25,26]. Because the analysis was conducted at the municipal level, the observed association represents a relationship between territorial characteristics and dengue incidence and cannot be extrapolated to individuals.

Taken together, the results indicate that dengue incidence in Bahia is a multidimensional phenomenon. The spatial distribution was not random, forming high-transmission clusters that varied in time and space. Socioeconomic and demographic factors were significantly associated with incidence, but their relationships are complex and, at times, counterintuitive, pointing to the influence of reporting bias and structural inequalities in access to health care, factors that must be considered when interpreting the data and formulating public health policies.

Limitations should be considered when interpreting these findings. First, the study relied on secondary surveillance data, which may be affected by underreporting, differences in case detection, and inconsistencies in information systems. Second, the ecological design is subject to ecological fallacy and does not permit individual-level causal or risk inferences. Third, socioeconomic and demographic indicators were primarily obtained from the 2010 Demographic Census, as more recent census data with municipal-level disaggregation were not available for the study period; consequently, changes in demographic characteristics, socioeconomic conditions, and urban infrastructure occurring between 2010 and 2024 may not have been fully captured, potentially resulting in temporal mismatch between the exposure indicators and dengue outcomes. Fourth, spatial regression was restricted to 2021 and 2024, selected as the years with the lowest and highest incidence rates, respectively; although this approach allowed comparison of contrasting transmission contexts, it limits assessment of how the associations between territorial characteristics and dengue incidence changed across the entire study period. Finally, climatic variables, vector density, human mobility, and other potentially relevant determinants were not included in the regression models and may account for part of the unexplained spatial variation.

Despite these limitations, the findings contribute to the understanding of the spatial and temporal distribution of dengue in Bahia by evidencing the presence of territorial clusters and associations with socioeconomic, demographic, and infrastructure indicators. From a public health perspective, the spatial identification of areas with sustained or emerging concentrations of dengue may support geographically targeted surveillance, prioritization of vector-control activities, and planning of interventions within the Brazilian Unified Health System (SUS).

Future studies could use more spatially disaggregated units, such as census tracts, and integrate climatic conditions, vector surveillance, and human mobility data. Such approaches could help clarify the mechanisms underlying the temporal changes and spatial clustering observed in this study while reducing some of the limitations associated with municipal-level ecological analyses.

## Conclusions

This study analyzed dengue incidence across the municipalities of Bahia between 2019 and 2024 using a multidimensional approach integrating temporal, spatial, socioeconomic, and demographic dimensions. Dengue incidence exhibited substantial temporal variation, with peaks in 2019, 2020, and 2024 and a marked decline in 2021, potentially influenced by changes in health care access, case detection, and surveillance during the COVID-19 pandemic. Spatial analysis demonstrated persistent positive spatial autocorrelation and geographically shifting High-High clusters, indicating that dengue incidence followed a nonrandom spatial pattern that varied across the state over time. The SAR model provided the best overall fit among the spatial regression models evaluated, and the observed associations with sewerage coverage, household income, and the proportion of the population classified as Black or mixed-race suggest that dengue incidence is related to multiple territorial dimensions rather than to a single determinant. These associations should be interpreted cautiously, as some may reflect differences in environmental conditions, health care access, diagnosis, and surveillance rather than differences in underlying individual-level risk.

The findings support a broader approach to dengue control that extends beyond vector control alone and considers the territorial conditions in which transmission occurs. Spatially identified areas of high incidence may serve as priorities for intensified surveillance and vector-control activities, while improvements in sanitation infrastructure and equitable access to health care and diagnosis may contribute to reducing territorial inequalities in disease detection and control. More spatially disaggregated studies incorporating climatic conditions, vector density, and human mobility could help clarify the mechanisms underlying the observed spatial and temporal patterns and strengthen the evidence base for territorially targeted interventions within the SUS.
